# Additive Manufacturing of Anatomical Poly(d,l-lactide) Scaffolds

**DOI:** 10.3390/polym14194057

**Published:** 2022-09-27

**Authors:** Dario Puppi, Gianni Pecorini, Gianluca Parrini

**Affiliations:** 1 BIOLab Research Group, Department of Chemistry and Industrial Chemistry, University of Pisa, UdR INSTM—Pisa, Via G. Moruzzi 13, 56124 Pisa, Italy; 2 Fabrica Machinale, Via Giuntini 13, Cascina, 56021 Pisa, Italy

**Keywords:** additive manufacturing, poly(lactide), fused deposition modeling, anatomical model

## Abstract

Poly(lactide) (PLA) is one of the most investigated semicrystalline polymers for material extrusion (MEX) additive manufacturing (AM) techniques based on polymer melt processing. Research on its application for the development of customized devices tailored to specific anatomical parts of the human body can provide new personalized medicine strategies. This research activity was aimed at testing a new multifunctional AM system for the design and fabrication by MEX of anatomical and dog-bone-shaped PLA samples with different infill densities and deposition angles. In particular, a commercial PLA filament was employed to validate the computer-aided design (CAD) and manufacturing (CAM) process for the development of scaffold prototypes modeled on a human bone defect. Physical-chemical characterization of the obtained samples by ^1^H-NMR spectroscopy, size exclusion chromatography (SEC), thermogravimetric analysis (TGA), and differential scanning calorimetry (DSC) demonstrated a small reduction of polymer molecular weight (~5%) due to thermal processing, as well as that the commercial polymer employed was a semicrystalline poly(d,l-lactide). Mechanical characterization highlighted the possibility of tuning elastic modulus and strength, as well as the elongation at break up to a 60% value by varying infill parameters.

## 1. Introduction

**Figure 1:**
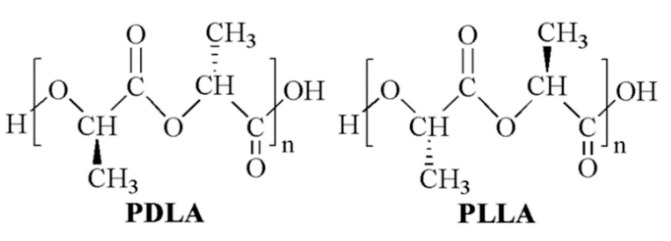
PLA chemical structure: poly(d-lactide) (PDLA) and poly(l-lactide) (PLLA).

Lactic acid (2-hydroxy propionic acid) is a chiral molecule consisting of two enantiomers. As a consequence, different stereoisomeric forms of poly(lactic acid) (PLA) are available, including poly(l-lactic acid) (PLLA), poly(d-lactic acid) (PDLA), poly(d,l-lactic acid) (PDLLA), and poly(l-lactic-co-d,l-lactic acid) (PLDLLA) (Figure 1) [1]. High molecular weight polymer batches are synthesized on an industrial scale through ring-opening polymerization of lactide, the cyclic dimer of lactic acid [2]. For this reason, commercial PLA is often referred to as poly(lactide). PLA is generally considered an environmentally friendly polymer because lactic acid is produced by microbial fermentation, typically in the L-form, and the resulting macromolecule, in turn, degrades back to lactic acid [3]. 

PLLA is a semicrystalline, thermoplastic polymer that undergoes a glass transition above room temperature (glass transition temperature, T_g_ ≅ 55–70 °C) [4]. As a consequence, and considering its hydrophobic nature, PLLA behaves as a brittle material when employed under physiological conditions (T = 37 °C, pH = 7.4). The crystallinity degree of PDLLA is decreased by increasing the D-isomer content until a complete amorphous morphology is obtained for molar percentages higher than 10–20%. As a consequence, PDLLA typically shows a lower T_g_ in the range of 50–60 °C, higher flexibility, and faster biodegradation than PLLA [5]. PLA is degraded in the human body mainly through nonspecific hydrolytic scission of ester bonds into oligomers and monomeric acids, which are metabolized through the tricarboxylic acid cycle and then excreted from the body in the form of carbon dioxide and water [6]. While the complete in vivo resorption of PLLA devices takes years [7,8], amorphous PDLLA can be completely resorbed by 12 months after implantation [9].

Thanks to their biocompatibility, suitable mechanical properties, and processing versatility, semicrystalline PLLA and PDLLA have been widely investigated in the biomedical field resulting in a range of biodegradable products currently available in the market for clinical use, such as suture reinforcements and anchors, meniscal darts, devices for osteosynthesis, and orthopedic fixation [10,11]. In addition, the relatively low T_g_ of PLLA is exploited for intravascular stent implantation procedures based on balloon expansion [12]. A considerable amount of literature has also been published on amorphous PDLLA to exploit its faster biodegradation for the controlled release of bioactive molecules [13].

PLLA and PDLLA are widely employed in additive manufacturing (AM) techniques involving processing polymers as a melt or solution [14], such as material extrusion (MEX) based on melt or solution processing, powder bed fusion, and binder jetting [15]. MEX of PLA was first described in 2001 in an article focused on a pneumatic system for polymeric grains melt processing [16]. After that, a number of articles have demonstrated the possibility of applying technologies commercially, referred to as fused deposition modeling or fused filament fabrication, to process PLLA filaments also loaded with natural fibers or inorganic fillers [17,18]. For instance, the successful fabrication and processing of PLA filaments loaded with hydroxyapatite and chitosan [19] or cellulose nanofibrils [20] were recently demonstrated. Several PLA products in the form of filament tailored to MEX are currently available on the market and supplied with optimized processing protocols. However, in most cases, the composition and stereoisomeric form of the supplied PLA batch is not specified. This is a critical aspect considering the great interest of the scientific community in AM of PLA for bone regeneration [21,22] and breast reconstruction [23], as well as for the production of custom-made oral dosage pharmaceutical forms or implantable drug-releasing systems [24]. Indeed, besides the obvious relationship between polymeric material composition and its biocompatibility, polymer crystallinity degree and molecular weight significantly affect the biodegradation rate of the resulting scaffold, as well as other properties, such as the mechanical behaviour [25,26]. 

The aim of this study was the design, fabrication, and characterization of customized PLA samples tailored to biomedical applications by means of a novel AM apparatus suitable for the employment of different techniques, i.e., solution- and melt-based MEX approaches, possibly integrated with electrospinning. As described in a recent article [27], this AM machine was already employed to fabricate tissue engineering scaffolds made of microbial polyester-based blends by means of computer-aided wet-spinning, a processing technique involving the extrusion and layer-by-layer deposition of a polymeric solution or suspension directly into a coagulation bath [28]. The present article deals with testing the melt-extrusion configuration of the machine through a computer-aided design (CAD) and manufacturing (CAM) process, enabling the fabrication of anatomically-shaped and clinically-sized physical models made of a commercial PLA (Sigma Aldrich), as well as relevant porous scaffolds for tissue regeneration. Additively manufactured PLA samples with a dog-bone shape tailored to tensile mechanical test, as well as a tuneable porous structure, were also fabricated and characterized by scanning electron microscopy (SEM) and uniaxial mechanical test. The fabricated samples were also characterized by proton nuclear magnetic resonance (^1^H-NMR), size exclusion chromatography (SEC), thermogravimetric analysis (TGA), and differential scanning calorimetry (DSC) to investigate the effect of melt processing on polymer molecular structure, as well as the stereoisomeric form of the employed PLA.

## 2. Materials and Methods

### 2.1. Materials

PLA (3D Printing filament; diameter: 1.75 mm; density: 1.24 g/cm^3^; tensile modulus: 65.5 MPa; vicat point: 55–60 °C; melting point: 144 °C), chloroform (CHCl_3_) for HPLC and deuterated chloroform (CDCl_3_) were bought from Sigma Aldrich (Milan, Italy) and used as received.

### 2.2. Additive Manufacturing

**Figure 2:**
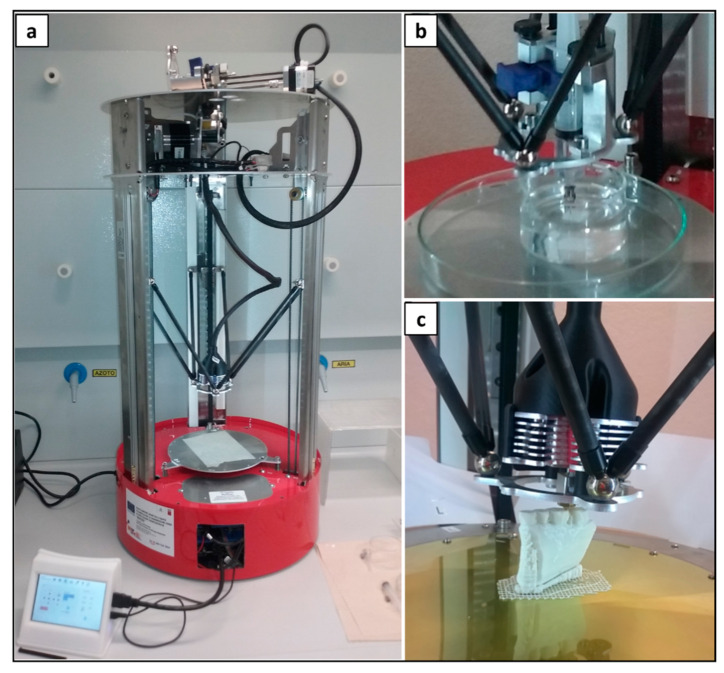
AM system with switchable extrusion heads: representative pictures of (**a**) the machine wired to the local touch screen control unit, (**b**) melt-extrusion head, and (**c**) solution-extrusion head.

Samples were fabricated by means of a multifunctional AM machine prototype (Fabrica Machinale S.r.l, Pisa, Italy) designed to process polymeric materials through either melt- or solution-based MEX by changing the operating head (Figure 2). As previously described in detail, the machine is equipped with six carbon bars terminating with magnetic spheres that are connected to the extrusion head, enabling its easy manual substitution.

Samples fabrication was carried out by employing optimized processing parameters in terms of infill angle, infill density, layer height (d_z_), infill speed, extruder T, and bed T. Anatomical and dog-bone shape samples were fabricated by processing customized digital models and G-code files. After printing, the samples were collected and stored in a desiccator before characterization.

### 2.3. Scanning Electron Microscopy (SEM)

Samples were analyzed by means of SEM (JEOL LSM 5600LV, Tokyo, Japan) after platinum sputter coating. Micrographs of sample top-view and cross-section were acquired at different magnifications. The arithmetical mean roughness (Ra) of the polymer matrix surface was estimated by processing top-view micrographs with Image J software (SurfCharJ_1q plugin).

### 2.4. Proton Nuclear Magnetic Resonance (^1^H-NMR)

^1^H-NMR spectroscopy was performed on a JEOL YH400 MHz spectrometer (JEOL Ltd., Arkishima, Tokyo, Japan). The spectra were recorded on a 1.6% *w*/*v* polymer solution in CDCl_3_ at 25 °C.

### 2.5. Size Exclusion Chromatography (SEC)

SEC analysis was carried out employing a liquid chromatograph PU-2089Plus (Jasco, Milan, Italy) with two columns PL gel 5_1 Mixed-D and a refractive index detector PI-2031 (Jasco, Lecco, Italy). Samples were dissolved in CHCl_3_ (0.5% *w*/*v*) and eluted in CHCl_3_ at a flow rate of 1 mL min^−1^. Number average molecular weight (M_n_), weight average molecular weight (M_w_), and polydispersity index (PI) were obtained. The instrument was calibrated by using polystyrene standard samples (range 0.5–300 kDa).

### 2.6. Thermogravimetric Analysis (TGA)

TGA was carried out using a Q500 instrument (TA Instruments, Milan, Italy) in the temperature range of 30–700 °C, at a heating rate of 10 °C min^−1^, and under a nitrogen flow of 60 mL min^−1^. The temperature corresponding to the maximum value of the peak on TGA first derivative thermograms (T_max_), as well as the temperature at which the sample starts to degrade (T_onset_), were recorded. 

### 2.7. Differential Scanning Calorimetry (DSC)

DSC analysis was carried out using a Mettler DSC-822 instrument (Mettler Toledo, Milan, Italy) by employing a heating and cooling rate of 10 °C min^−1^ in the temperature range of 25 to 200 °C, under a nitrogen flow rate of 80 mL min^−1^. The glass transition temperature (T_g_) was determined at the inflection point, and the melting temperature (*T_m_*) as the minimum of the endothermic peak in the first and the second heating cycles. The weight fraction of the d-lactide repeating unit in the macromolecular chain was calculated according to the following equation [29]:

(1) $$T_{m}\approx 175-3000\;w$$

where *w* is the fraction of meso-lactide repeating unit (i.e., an l-lactic acid and an d-lactic acid sequence) in the macromolecular structure.

### 2.8. Mechanical Characterization

Uniaxial tensile properties of dog-bone-shaped samples were evaluated at room temperature with an Instron 5564 instrument (Norwood, Instron, MA) by following ASTM standards D 1708-93 [30] and D 882-91 [31]. Five replicates for each kind of printed sample (38 × 15 mm^2^ overall size, 5 × 22 mm^2^ in the gage area; average thickness smaller than 1 mm and measured with a micrometer) were stretched to the breaking point at room temperature and ambient humidity, under a constant crosshead displacement of 4 mm min^−1^. Tensile stress-strain curves, elastic modulus, and stress and strain at break were obtained from software recording data (Merlin).

### 2.9. Statistical Analysis

Data are reported as mean ± standard deviation. Data were processed by two-way analysis of variance (ANOVA) and Tukey test for post hoc analysis. Differences were considered significant for a *p*-value < 0.05.

## 3. Results and Discussion

### 3.1. Digital Modelling and Fabrication

**Figure 3:**
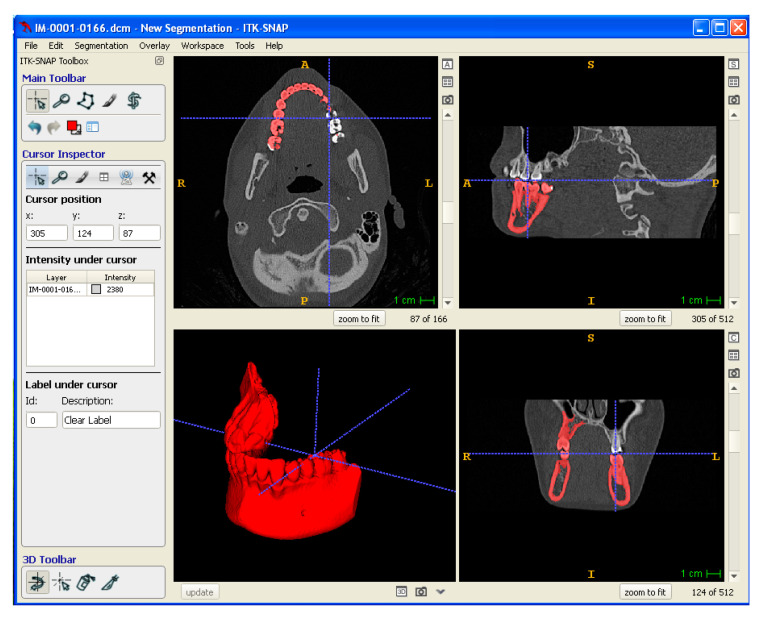
Digital modeling: segmentation of DICOM data to obtain a 3D Model exported in STL format.

Medical imaging data of a mandibular tissue defect obtained by computer tomography (CT) were processed by means of CAD/CAM software to physically reproduce anatomical models and porous scaffolds. In particular, CT data were exported in Digital Imaging and Communications in Medicine (DICOM) format. ITK-SNAP software was used for the segmentation of the mandibular area. Segmentation was performed using a semi-automatic method, density-related volume growing. After segmentation, the 3D model so obtained was exported into a standard tessellation language (STL), which was then sliced into layers, originating a slice file (SLI) (Figure 3). A numerical control (NC) programming language file written in G-code was generated and loaded digitally into the machine to drive the motion of the fabrication process.

**Figure 4:**
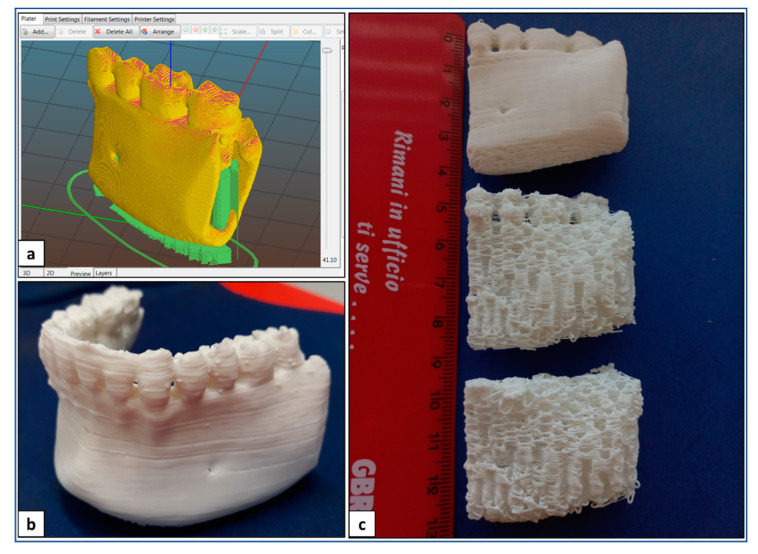
AM of PLA: (**a**) digital model of a lay-down pattern; (**b**) representative picture of lower jaw physical model; (**c**) representative picture of physical models and porous scaffolds of madibular bone segment.

Optimized design and processing parameters for PLA sample fabrication are summarized in Table 1. In particular, these parameters were applied to fabricate anatomical physical models of lower jaw parts, as well as anatomical scaffolds customized on the mandibular bone segment defect and designed with an infill density of 100 or 60% (Figure 4).

**Figure 5:**
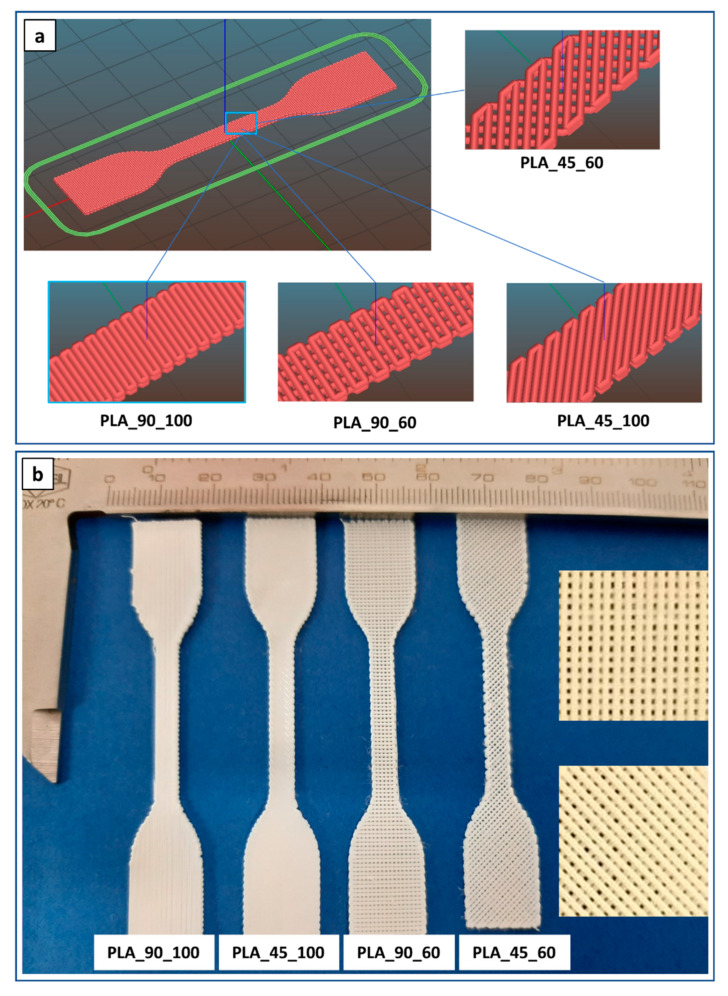
Dog-bone-shaped PLA samples: (**a**) lay-down digital models and (**b**) representative pictures (insets are high magnification details of 60% infill density specimens) of samples with different infill angles and density.

In addition, the optimized parameters were applied to fabricate dog-bone-shaped samples designed with infill angle and density of 90° and 100% (PLA_90_100), 45° and 100% (PLA_45_100), 90° and 60% (PLA_90_60), or 45° and 60% (PLA_45_60) (Figure 5).

### 3.2. Morphological Characterization

**Figure 6:**
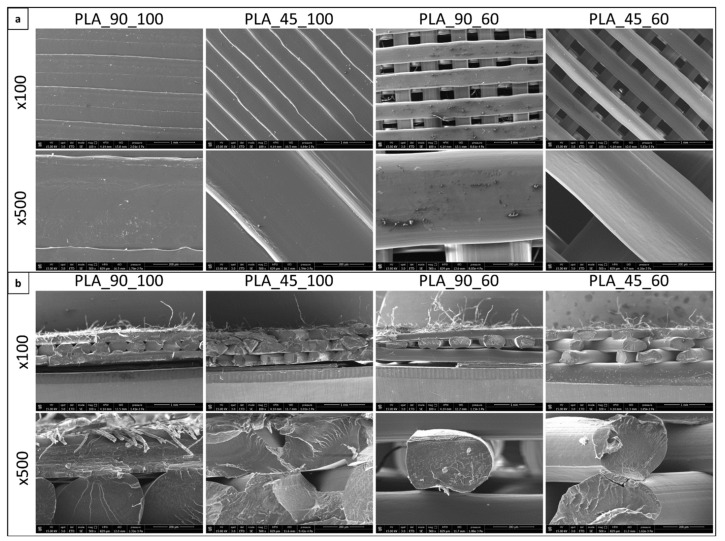
SEM analysis: (**a**) top-view and (**b**) cross-section of dog-bone-shaped PLA samples with different infill density and angle (magnification 100× and 500× in both cases).

The developed dog-bone-shaped samples were characterized to analyze the polymer microstructure and how this can be related to the resulting mechanical properties. Samples fabricated with theoretical 100% infill density were characterized by marked fusion at the filament–filament contact points, even if a memory of filament cylindrical shape is evident (Figure 6). In the case of samples designed with a 60% infill density, high filament alignment along the lay-down direction was observed. The microfibers attached to the filament surface observed in some micrographs are residual filter paper, which was used to optimize the attachment of the first printed layer to the deposition platform.

Arithmetical average roughness (Ra) values of the characterized samples are shown in Table 2. The obtained data are comparable to those reported in the literature relevant to MEX of PLA [32,33]. Statistical analysis showed that PLA_90_100 samples were characterized by the significantly lowest Ra value, while the PLA_45_100 sample had a significantly lower value than the PLA_45_60 sample. Surface roughness of additively manufactured samples is typically considered a disadvantage because it may cause stress concentration, reducing materials strength [34]. For this reason, post-processing treatments, such as surface polishing, are applied to reduce surface roughness and enhance the performance of the fabricated part in the working environment. On the contrary, surface roughness is often considered an advantage for biomaterials since it plays a significant role in cell colonization by favoring the adsorption of adhesion proteins and the subsequent cell adhesion, as well as the mechanical stability of implants in host tissues [35]. As a consequence, a large body of literature in the field of tissue engineering and regenerative medicine has investigated surface engineering at the micro- and nanoscale as a means to control cell behavior, including cell adhesion, orientation, migration, and differentiation [36].

PLA_45_60 samples were further characterized as representative additively manufactured specimens for their physical–chemical and thermal properties, as described in the following sections.

### 3.3. Physical-Chemical Characterization

**Figure 7:**
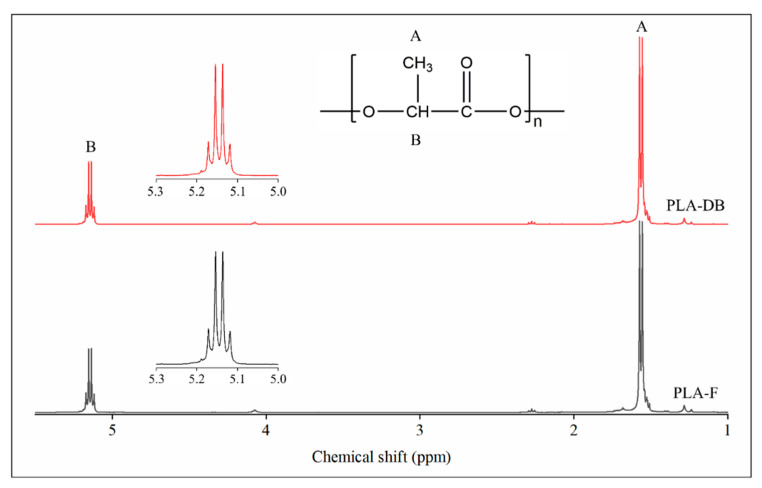
^1^H-NMR spectroscopy: spectra of the filament PLA-F (black) and the dog-bone-shaped sample PLA-DB (red).

^1^H-NMR analysis was carried out on CDCl_3_ solutions of PLA filament (PLA-F) and PLA_45_60 dog bone (PLA-DB) specimens. In both cases, the spectra showed a signal at 1.56 ppm relative to the protons of the methyl group of the lactide units and a signal centered at around 5.15 ppm associated with the resonance of the protons of the methine group (Figure 7). The signal at 5.15 ppm consists of a quartet revealing the presence of different sequences of stereocenters constituting the polymer chain backbone. Since lactic acid has two possible configurations, R and S, depending upon the arrangement of substituents around the chiral carbon, three possible configurations of its cyclic dimer lactide can occur, i.e.,  RR (d-lactide), SS (l-lactide), and RS (meso-lactide). According to Zell et al. [37], the observed NMR resonances can be assigned to various combinations of “*i*” isotactic pairwise relationship (-RR- and -SS-) and “*s*” syndiotactic pairwise relationship (-RS- and -SR-). In particular, the signal at 5.17 can be related to *iis*/*sii* sequences, the two signals at 5.15 and 5.14 to *ii* sequences, while the signal at 5.12 ppm to *isi* sequences. The integrated intensity of each resonance is directly proportional to the percentage of stereocenters producing that signal. The *iis*/*sii* resonance at 5.17 ppm gave an integrated intensity of 17%, while the *isi* resonance at 5.12 ppm gave an integrated intensity of 15%. 

SEC analysis was carried out to determine the molecular weight of the starting PLA and its decrease after processing by MEX. As summarized in Table 3, M_n_ and M_w_ of the starting filament (PLA_F) were 86,600 and 124,600 g/mol, respectively. Those values fall in the range of the optimal PLA molecular weight for MEX processing to achieve suitable melt viscosity during the extrusion process, i.e., 50,000–140,000 g/mol [38].

PLA M_n_ e M_w_ were reduced after processing by about 4 and 5%, respectively. As previously described in the literature [39], PLA can be susceptible to degradation during thermal processing. PLA processed employing AM techniques based on extruding polymer granules can be subjected to a higher decrease in molecular weight, such as an M_n_ decrease of about 35% [40]. However, it should be considered that in the case of MEX, the polymer has already been melt-extruded before 3D printing to prepare a filament.

### 3.4. Thermal Characterization

**Figure 8:**
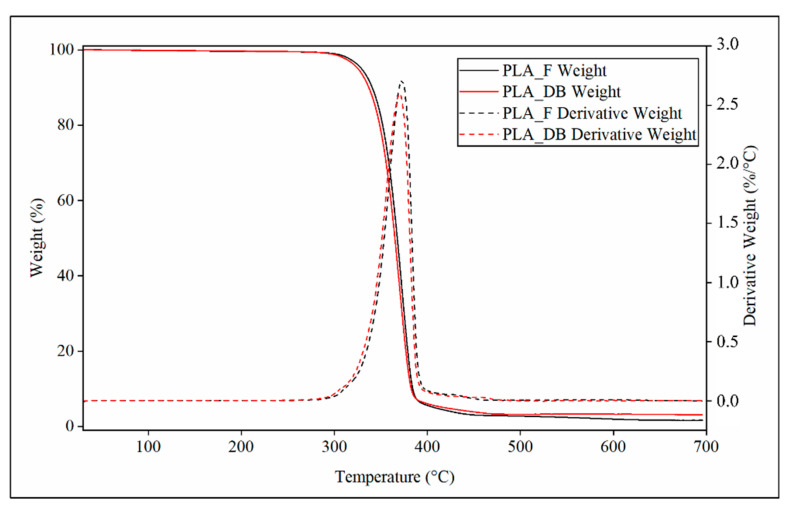
TGA characterization: representative thermograms relevant to profile of weight (continuous line) and derivative weight (dotted lines) of PLA_F (black curves) and PLA_DB sample (red curve).

Representative TGA thermograms of PLA-F and PLA-DB relevant to the variation of weight or derivative weight vs. temperature are reported in Figure 8. 

Both samples showed a single phenomenon of weight decrease associated with a peak of the derivative weight curve. PLA_DB sample showed an average onset temperature (T_onset_) of about 347 °C and a temperature of maximum degradation rate (T_max_) of about 370 °C, both values lower than those of PLA_F (T_onset_ = 350 °C and T_max_ = 372 °C) (Table 4). These small but statistically detectable differences can be related to the reduction of the polymer molecular weight due to MEX processing, as demonstrated by SEC analysis.

The traditional mechanism ascribed to the degradation of PLA by pyrolysis is a complex phenomenon that predominantly consists of random main chain scission and unzipping depolymerization reactions [41]. The random degradation involves intramolecular and intermolecular transesterifications, cis-elimination, and hydrolysis. The dominant reactions of thermal degradation are the intramolecular and intermolecular transesterifications leading to cyclic oligomers of lactic acid and lactide. Unzipping depolymerization is accelerated when terminal hydroxyl groups become more concentrated.

**Figure 9:**
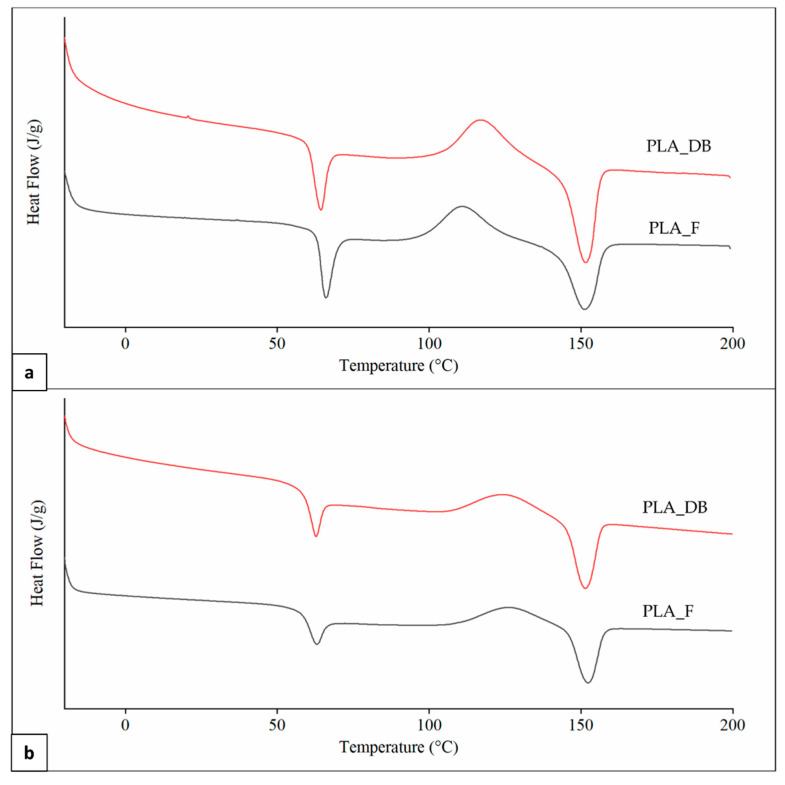
DSC analysis: representative thermograms of PLA_F and PLA_DB samples relevant to (**a**) first and (**b**) second heating.

Representative DSC thermograms of PLA_F and PLA_DB samples relevant to the first and second heating scans are reported in Figure 9. An inflection point associated with the polymer glass transition temperature (T_g_) in the range from 61 to 64 °C, partially overlapped with an enthalpic relaxation peak, was evident in all thermograms. An exothermic peak centered at a temperature (T_cc_) in the range of 110–126 °C and related to PLA cold crystallization, as well as an endothermic peak centered at around 151 °C (T_m_) as a result of the polymer crystalline phase melting were also observed in all curves. 

A significantly higher T_g_ in the first heating scan in comparison to the second one was obtained for both samples (Table 5). This is likely due to a higher orientation degree of the macromolecular chains as a result of melt extrusion, reducing polymer fractional free volume and molecular mobility [42]. Samples heating during DSC analysis erases polymer thermal history, making the macromolecular chains lose their preferential orientation and reducing T_g_ [43]. First heating thermograms were also characterized by a lower T_cc_, as well as higher cold crystallization enthalpy (ΔH_cc_) and crystallinity degree (χ), possibly because of an effect of macromolecular orientation on the crystallization process. Significant differences in T_g_ and T_cc_ values between PLA_F and PLA_DB are related to different effects on the macromolecular conformation of the two relevant processing techniques (i.e., filament screw-extrusion and MEX, respectively) [44]. Indeed, no significant differences between the thermal parameters of the two samples were detected in the second heating scan after thermal history erasing. This also demonstrates that the recorded molecular weight reduction after MEX processing does not affect polymer morphology.

As previously mentioned, PLLA is a semicrystalline polymer with a T_m_ of around 175 °C. Small percentages of d-lactide repeating units can significantly reduce polymer crystallinity degree and T_m_ down to 130 °C, and a fully amorphous morphology is typically obtained in the case of l-lactide percentage lower than 90% [38]. The T_m_ values recorded during DSC analysis corroborate the results achieved by ^1^H-NMR spectroscopy regarding the presence of d,l-lactide repeating unit in the macromolecular backbone. In particular, on the basis of T_m_ of PLA_F obtained from the second heating thermograms, the fraction of d,l-lactide repeating unit was estimated to be 7.8 ± 0.1% by employing the equation (Equation (1)) proposed by Auras et al. [29].

### 3.5. Mechanical Characterization

**Figure 10:**
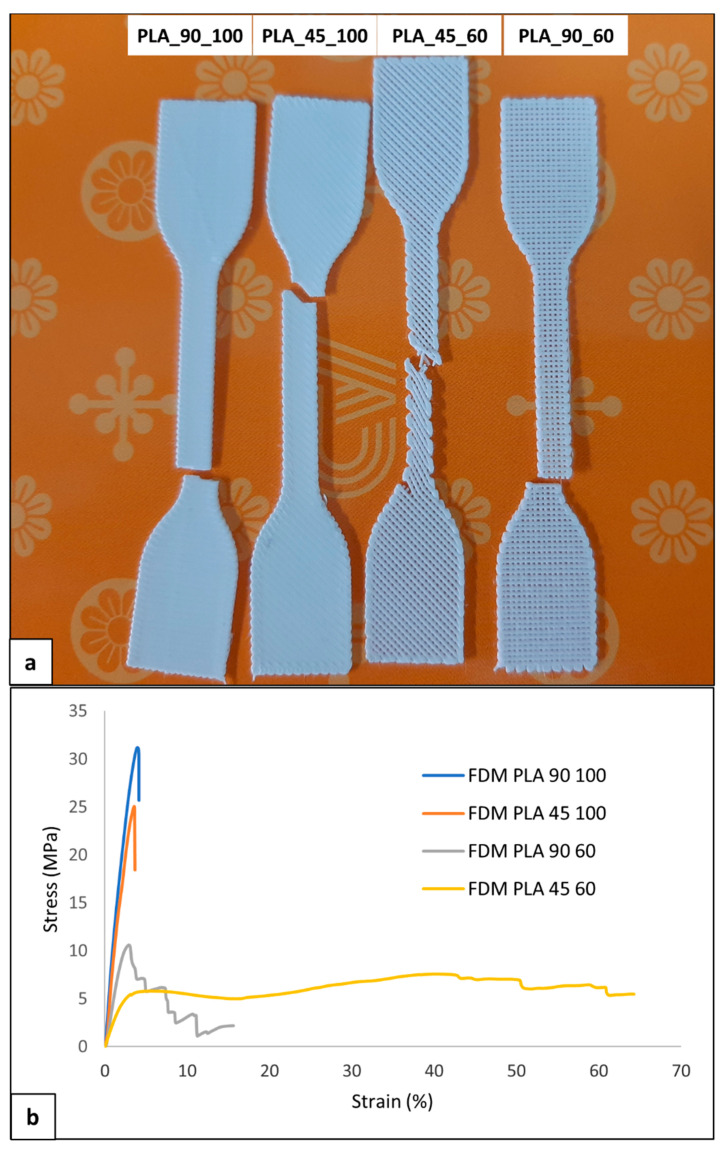
Mechanical characterization of PLA samples: (**a**) representative picture of broken samples and (**b**) stress-strain curves of the four kinds of developed samples.

Uniaxial tensile characterization of the developed dog-bone-shaped samples was carried out. The four kinds of the sample had different visual breaking behavior and morphology (Figure 10a), as well as different profiles of the stress-strain curve (Figure 10b). 

Samples with 100% infill density were characterized by elastic deformation and then breaking shortly after the yielding point without a marked plastic deformation at strain values lower than 5% (Table 6). Samples with an infill density of 60% also demonstrated plastic deformation. PLA_90_60 samples showed, in some cases, a sequential breaking of the single filaments parallel to the sample’s longitudinal axis. This visual breaking behavior was reflected in a multistep stress drop in the σ-ε curve. Regarding PLA_45_60 samples, a reorientation of the filament during deformation with a gradual decrease in the filament axis angle relative to the sample longitudinal axis was evident. This macrostructural reorganization resulted in a plateau region that extended up to relatively high values of elongation at break (ε_break_).

The 100% infill density samples showed a significantly larger tensile modulus and strength (E > 1 GPa, σ_max_ > 30 MPa) than 60% infill density samples (Table 6), as a consequence of the larger effective resistant area related to a dense, continuous polymeric matrix. On the other hand, PLA_45_60 samples had a significantly larger ε_break_ (68.2 ± 15.1%) than the other samples.

Different published articles have reported that the tensile modulus and strength of MEX-manufactured PLA samples (1–4 GPa and 15–75 MPa, respectively) are comparable to those of PLA films made by solvent casting or compression molding [45,46,47]. In particular, Chacón et al. [48] recently demonstrated that the tensile and three-point bending properties of PLA can be significantly tuned by acting on build orientation, layer thickness, and feed rate. Other parameters, such as extrusion head and bed temperature, as well as lay-down pattern, were demonstrated to play a significant role in the resulting material mechanical response [49,50]. The present experimental characterization contributes to this research trend by highlighting that infill density and lay-down pattern are two key parameters exploitable for PDLLA tensile properties tuning. In particular, the combined effect of 60% infill density and 45° infill angle is reflected in much higher tensile deformation in comparison to what conventional achieved with semicrystalline PLA (ε_break_ < 10%).

## 4. Conclusions

The developed AM system prototype is well suited to the design and fabrication of anatomically-shaped, clinically-sized PLA scaffolds for bone regeneration. Experimental protocols for investigating the composition and morphology of commercial PLA filaments were optimized by combining different characterization techniques. In particular, ^1^H-NMR and DSC analyses enabled the identification of the polymer stereoisomeric form and morphology, showing that the employed polymer was a semicrystalline PDLLA. In addition, TGA and SEC characterization highlighted a limited polymer thermal degradation due to AM (≈4% reduction of M_n_). Another significant finding of this study is that the elongation at break of PLA samples can be markedly varied by acting on AM lay-down pattern and infill density. More research is needed using other printing parameters to assess whether material flexibility and elongation at break can be further tuned. Moreover, a natural progression of this work is the investigation of biodegradable polymers not yet available on the market in the form of filaments (e.g., microbial polyhydroxyalkanoates [51]) for the development of scaffolds with an anatomical shape and customized porous structure.

## Figures and Tables

**Table 1:** Optimized parameters for PLA processing.

Parameter	Optimized Value
Infill angle	90° or 45°
Infill density	60 or 100%
Nozzle diameter	400 μm
Layer height (d_Z_)	300 μm
Infill speed	10 mm/s
Extruder T	210 °C
Bed T	40 °C

**Table 2:** Data relevant to surface roughness analysis.

Sample	Ra (μm)
PLA_90_100	1.95 ± 0.30 ^a^
PLA_45_100	4.59 ± 0.65 *
PLA_90_60	6.13 ± 0.80
PLA_45_60	6.67 ± 0.97 *

Data are reported as mean ± standard deviation (n = 3). ^a^ The value marked with this symbol is significantly lower than the others. * Data marked with the same symbol are statistically different (*p* < 0.05).

**Table 3:** Data relevant to SEC analysis.

Sample	M_n_ (g/mol)	M_w_ (g/mol)	PI
PLA_F	86,600 ± 400 *	124,600 ± 500 **	1.439 ± 0.001 ***
PLA_DB	83,000 ± 300 *	118,000 ± 500 **	1.422 ± 0.002 ***

Data are reported as mean ± standard deviation (n = 3). *, **, *** Data marked with the same symbol are statistically different (*p* < 0.05).

**Table 4:** Data relevant to TGA analysis.

	T_onset_ (°C)	T_max_ (°C)
PLA_F	350.1 ± 0.4 *	372.4 ± 0.1 **
PLA_DB	346.9 ± 0.5 *	369.8 ± 0.4 **

Data are reported as mean ± standard deviation (n = 3). *, ** Data marked with the same symbol are statistically different (*p* < 0.05).

**Table 5:** Data relevant to DSC analysis.

	Sample	T_g_ (°C)	T_cc_ (°C)	ΔH_cc_ (J/g)	T_m_ (°C)	χ (%)
First heating	PLA_F	64.3 ± 0.2 ^a^	110.7 ± 0.4 ^a^	21.4 ± 2.0 *	150.9 ± 0.2	28.6 ± 0.6 *
	PLA_DB	62.0 ± 0.3 ^a^	117.3 ± 0.8 ^a^	22.8 ± 2.0 *	151.5 ± 0.5	27.9 ± 2.5 *
Second heating	PLA_F	61.0 ± 0.2 *	126.6 ± 0.5 *	10.5 ± 0.8 **	151.9 ± 1.0	14.0 ± 0.5 **
	PLA_DB	60.9 ± 0.6 *	126.2 ± 1.9 *	10.7 ± 0.9 **	151.6 ± 0.3	13.0 ± 2.6 **

Data are reported as mean ± standard deviation (n = 3). *, ** Data marked with the same symbol are statistically different from the others but not between themselves (*p* < 0.05). ^a^ Data marked with this symbol are statistically different from all the others (*p* < 0.05).

**Table 6:** Tensile mechanical parameters of PLA samples.

Sample	E (GPa)	σ_max_ (MPa)	ε_break_ (%)
PLA_90_100	1.3 ± 0.1 *	33.1 ± 3.4 *	4.1 ± 0.6
PLA_45_100	1.1 ± 0.2 *	32.6 ± 8.5 *	4.9 ± 0.9
PLA_90_60	0.5 ± 0.1 **	9.6 ± 1.1 **	11.2 ± 4.6
PLA_45_60	0.3 ± 0.1 **	8.0 ± 1.4 **	68.2 ± 15.1 ^a^

Data expressed as mean ± st. dev. (n = 5). *, ** For a given parameter (E or σ_max_), values marked with the same symbol are significantly different to the others, but not between themselves. ^a^ Value significantly larger than the other values relevant to ε_break._

## Data Availability

The data presented in this study are available on request from the corresponding authors.
